# Innovation in Flow Cytometry Analysis: A New Paradigm Delineating Normal or Diseased Bone Marrow Subsets Through Machine Learning

**DOI:** 10.1097/HS9.0000000000000173

**Published:** 2019-02-22

**Authors:** Francis Lacombe, Benoît Dupont, Nicolas Lechevalier, Jean Philippe Vial, Marie C. Béné

**Affiliations:** 1 Hematology Laboratory, Bordeaux University Hospital, Pessac, France; 2 Beckman Coulter France S.A.S., Villepinte, France; 3 Hematology Biology, Nantes University Hospital, Nantes, France

Multiparameter flow cytometry (MFC) has become an undisputed method for the diagnosis and follow-up of hematopoietic malignancies through the analysis of leukocyte subpopulations.^1^ Although a lot of experience has been acquired in the routine application of this method, subjective approaches still remain the rule, responsible for the lack of standardization frequently perceived for this technique. The emergence of software allowing finally for unsupervised assessment of normal hematopoietic differentiation, a long-nurtured dream, finally rose from the development of mass cytometry (MC).^2^ Here we report how the application of such novel software, in combination with flow data analysis classical tools, allows for a better and original exploration of normal hematopoiesis pathways and, ultimately, disease and minimal residual disease (MRD) assessment.

A classical widely used representation of MFC is the CD45/side scatter (SSC) biparametric histogram upon which various subsets, identified through a series of supervised gates, can be backgated.^3^ In this type of representation, immature progenitors are typically low SSC intermediate CD45^+^ cells and maturation toward the granulocytic, lymphoid or monocytic lineages can be appreciated as a continuum. However, a more precise delineation of maturation subsets cannot be performed with these approaches relying on arbitrary thresholds never directly considering all simultaneously acquired parameters together. The separation of pathological subsets in disease is hampered by the same subjective appreciations, in spite of efforts at harmonization.^4,5^

The less supervised approach of principal component analysis (PCA) has confirmed the presence of more or less well-separated subsets. In whole bone marrow (BM), PCA can individualize immature and mature subsets. On selected pathological populations, PCA has been used to assign malignant cells to a specific lineage or type of lymphoproliferative disorder.^6,7^

The new software developed for MC provide bidimensional graphic representations of clusters delineated in a highly multidimensional space.^8^ The sophisticated technology of MC is, however, not yet adapted to routine analyses performed daily for the diagnosis and follow-up of hematological malignancies. Moreover, the software solutions developed for MC are time consuming and do not provide reproducible patterns.^8^

The FlowSOM program,^9^ initially designed for MC within the open access Bioconductor open-source R project, has been shown by its inventors to be efficient also for classical fluorescence MFC. This solution has been praised by the International Society for Applied Cytometry for its discriminative abilities and operator-friendly application.^10^ FlowSOM can be programmed to extract up to 100 nodes ordered in minimal spanning trees (MST). Applied to classical MFC, it can also, unlike MC, take into account the scatter properties (SSC) of the cells. Here we report how unsupervised FlowSOM analysis can guide in depth subsets identification by the combined use of a classical MFC software.

In a first step, four 10-color antibody combinations reported previously^11^ were applied to normal BM samples (Table S1, supplemental Digital Content). The latter had been obtained from adults without any hematological disorder during thoracic surgery, collected on EDTA-K, stained in a lysis no wash manner and acquired on a Navios instrument (Beckman Coulter, Miami, FL) according to Harmonemia recommendations.^5^ The flow cytometry standard (fcs) files obtained after acquisition of these normal BM samples with the 4 panels were merged and submitted to the unsupervised analysis of FlowSOM scripts, resulting in 4 reference MST.

New dedicated R scripts were developed to obtain a representation of the global unsupervised multiparametric analysis of each panel as an MST according to FlowSOM strategy and integrated in the classical MFC software Kaluza (Beckman Coulter). This tool was then used to further identify each of the MST subsets (ie, “nodes”) according to its whole immunophenotypic characteristics (mean fluorescence intensity, cell numbers, percentages).

As shown in Figure 1A, the CD45/SSC biparametric histogram of merged normal BM yielded 100 MST unsupervised subsets or “nodes,” highlighting the complexity of normal BM. Figure 1B shows how node-by-node analysis, with the classical tools of Kaluza, allowed to identify their immunophenotypic characteristics in reference patterns issued from the 4 panels tested, providing a refined objective delineation of BM differentiation pathways. One of the great strengths of the combination of FlowSOM MST unsupervised analysis and Kaluza specificities is that each node can be thoroughly dissected in a series of classical biparametric histograms. Figure 1C provides examples discriminating classical and nonclassical monocytes which appear as a single node^12^ and how 3 nodes of myeloid progenitors segregate the most immature subsets of CD34^+^/CD38^−^, CD34^+^/CD38^+^, and CD34^dim^/CD38^+^ cells.^13^ Indeed, sophisticated gating strategies have been reported so far to arbitrarily isolate these specific subsets.^5^ Here, the delineation is straightforward and comes directly from the unsupervised analysis, followed by operator-driven examination of cells’ characteristics.

**Figure 1:**
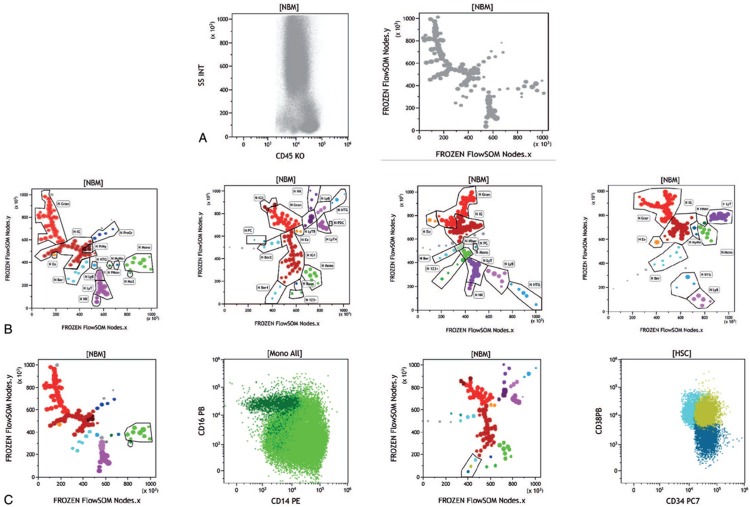
**Unsupervised delineation of human normal bone marrow.** (A) Left: CD45/SSC representation of 19 normal merged bone marrow (BM) samples stained with acute myeloid leukemia (AML) panel B and acquired according to Harmonemia recommendations.^5^ Right: minimal spanning trees (MST) obtained after unsupervised multidimensional (11 dimensions) analysis by FlowSOM of the same population of 19 normal BM samples. Legend: 123+ = undefined CD123 population, Baso = basophils, Ber = bermudes, Ber1= bermudes 1, Ber2 = bermudes 2, Eo = eosinophils, Gran = granulocytes, HTG = hematogones, IG = immature gran (IG1, IG2), LyB = B cells, LyT = T cells (LyT CD4; LyT CD8), Mo = monocytes, Mo3 = nonclassical monocytes, MonoBer = monocyte progenitors, MyMo = myelomonocytes, NK = NK cells , PC = plasma cells, pDC = plasmacytoid DC, PrMo = promonocytes, PrMy = promyelocytes, ProGR = progenitor granulocytes. (B) Four different MST were obtained with the 2 AML and 2 acute lymphoblastic leukemia panels explored after merging the normal bone marrows stained with these antibodies. Node-by-node exploration of immunophenotypic characteristics of each isolated cell subset allowed to assign node clusters to specific hematopoietic populations. (C) Left: Focus on the monocytic cluster (light green) and the isolated node dubbed Mo3 (dark green) on the colored MST of AML-A stained normal merged BM. The biparametric representation of these gates shows the superimposition yet clear identification of nonclassical monocytes^12^ (CD14^dim^, CD16^+^) segregated by FlowSOM as Mo3. Right: colored MST of AML-B stained normal merged BM, with a focus on the tree nodes of immature progenitors (bermudes).^5^ The biparametric CD34/CD38 histogram shows the superimposition of the 3 subsets^13^, respectively, CD34^+^CD38^−^ (dark blue), CD34^+^CD38^+^ (gold), and CD34^lo^CD38^+^ (cyan). In this classical representation, manual gating would be highly subjective while whole FlowSOM clearly delineates 3 nodes.

Based on these new reference BM display patterns, the corresponding FlowSOM R-script was applied to acute myeloid leukemia (AML) BM samples investigated at diagnosis and follow-up with the same AML panels in the course of routine laboratory analysis. One example is shown in Figure 2, where the leukemic population appeared as a single cluster on a classical CD45/SSC cartography. FlowSOM unsupervised analysis allowed to observe, in the expected nodes, elements of residual hematopoiesis. Color-backgating of the CD45/SSC leukemic cluster highlighted several nodes on the MST, providing a direct visualization of AML immunophenotypic heterogeneity. Concomitant analysis of a follow-up sample from the same patient allowed to clearly see which of these subclones had disappeared or survived upon chemotherapy (see figure legend).

**Figure 2:**
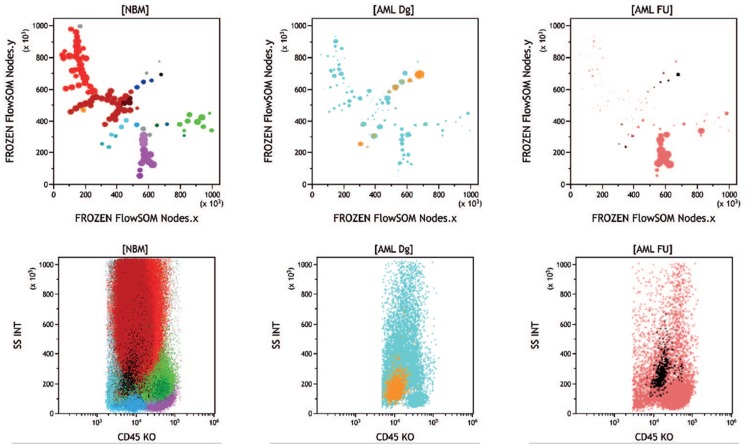
**Unsupervised analysis of diagnosis and follow-up samples from an AML patient.** Normal merged BM and patient's samples stained with the AML-A antibody combinations; the middle top panel shows the diagnosis sample; the top right panel shows a follow-up sample with measurable residual disease. The bottom panels display the CD45/SSC patterns of the respective MST. The diagnosis sample shows, by comparison with the reference normal BM MST, a severe disappearance of granulocytic maturation and near complete disappearance of monocytic maturation, also identifiable, but more roughly, in the corresponding CD45/SSC histogram. The blasts cluster (13.7%), which appears as a single population in the CD45/SSC histogram is in fact segregated in several nodes in the corresponding MST (golden nodes). In the follow-up sample, the lymphoid compartment is still predominant. Although in smaller proportion (1.44%), remaining blasts are in the same position as at diagnosis (black nodes). Of note, the same nodes are present in normal bone marrow but represent only 0.18% of leukocytes and appear as a scattered population on the CD45/SSC scattergram. This sample is representative of 20 patients whose diagnosis and follow-up have been performed with the same panel and successfully submitted to FlowSOM analysis (data not shown). BM = bone marrow, MST = minimal spanning trees.

The same strategy can be successfully applied to acute lymphoblastic leukemia (data not shown) where the tracking of MRD has been largely published and is much easier than in AML.

In summary, we report here on the bioinformatics innovation of an original combination of available software, likely to be universally implemented for a new, objective and comprehensive vision of normal and diseased BM. This opens the field of countless applications, through the use of different panels, specifically adapted to given subsets or diseases. The already achieved obtention of reference patterns of normal BM allows for an instant visual identification of anomalies when a diseased sample tested with the same panel is displayed concomitantly. The latter can then be finely analyzed, if needed, node by node, for an unbiased appreciation. More accurate results are thus to be expected in a near future for the benefit of hematology patients, through a more precise unsupervised definition of cell subsets.

## Supplementary Material

Supplemental Digital Content
