# Supplementary material for: Innovation in Flow Cytometry Analysis: A New Paradigm Delineating Normal or Diseased Bone Marrow Subsets Through Machine Learning
Source: Hemasphere. 2019 Feb 22;3(2):e173. doi: 10.1097/HS9.0000000000000173 (PMC6746040; doi:10.1097/HS9.0000000000000173)
Supplement: Supplemental Digital Content [file hs9-3-e173-s001.docx]

**Table 1.** Antibody combinations used here from panels consensually developed for the immunophenotyping of acute myeloblastic (AML) or lymphoblastic (ALL) leukemia^15^

|  | **FITC** | **PE** | **ECD** | **PC5.5** | **PC7** | **PC** | **Alexa 700** | **Alexa 750** | **Pacific Blue** | **KromOrange or V500** |
| --- | --- | --- | --- | --- | --- | --- | --- | --- | --- | --- |
| AML-A | CD65/15 | CD14 | CD13 | CD33 | CD34 | CD117 | CD7 | CD11b | CD16 | CD45 |
| AML-B | CD64 | CD10 | CD4 | CD33 | CD34 | CD123 | CD56 | CD19 | CD38 | CD45 |
| ALL-A | CD58 | CD10 |  |  | CD34 | CD123 |  | CD19 | CD38 | CD45 |
| ALL-B | CD81 | CD10 | CD13 | CD33 | CD34 | CD15 | CD22 | CD19 | CD20 | CD45 |
